# Suture Anchor Repair for Distal Patellar Tendon Avulsion in Tibial Tubercle Fractures: A Technical Description and Report of Two Cases

**DOI:** 10.7759/cureus.54696

**Published:** 2024-02-22

**Authors:** Robert Wood, Jacqueline Krumrey, Kathryn Palomino, Megan Hicks

**Affiliations:** 1 Orthopedics, Good Samaritan Regional Medical Center, Corvallis, USA; 2 Orthopedics and Traumatology, Good Samaritan Regional Medical Center, Corvallis, USA; 3 Pediatric Orthopedics, Legacy Emmanuel Children's Hospital, Portland, USA; 4 Biology, Virginia Tech Carilion School of Medicine, Blacksburg, USA

**Keywords:** orthopedics, trauma and orthopedics, sports injury surgery, youth sports, pediatric sports injury, knee surgery sports traumatology and arthroscopy, knee surgery outcomes, pediatric orthopedic surgery, orthopedic surgery, pediatric orthopedics

## Abstract

Tibial tubercle avulsion fractures, though rare, pose a significant challenge in pediatric orthopedics, particularly in athletic adolescents. For nondisplaced fractures, conservative treatment involves the use of braces or casts, while displaced fractures necessitate operative intervention, often through screw fixation. Concomitant soft tissue injuries should also be identified and addressed operatively to ensure complete repair of the extensor mechanism. This paper introduces a method for open reduction and internal fixation (ORIF) of tibial tubercle fractures with suture anchor repair of the distal patellar tendon avulsion. Two case examples of 14-year-old males with displaced fractures undergoing this procedure are presented. After standard screw fixation of the displaced fragment was performed, a single suture anchor was placed into the tibia and an onlay tension slide technique was utilized to secure the distal patellar tendon avulsion. Both patients underwent immobilization and protected weightbearing for four weeks with physical therapy initiated at six weeks. At four months postoperatively, both patients had returned to competitive sports without issue. The described technique may represent a reliable and reproducible method for addressing the distal patellar tendon avulsion component of tibial tubercle fractures. Its biomechanical advantages contribute to the complete repair of the extensor mechanism, enabling a successful return to competitive athletics without hardware complications.

## Introduction

Tibial tubercle avulsion fractures, although rare, present a unique challenge in the realm of pediatric orthopedics, demanding a meticulous understanding of their mechanisms, classification, and comprehensive assessment. Incidence rates typically quoted as less than 1% of all pediatric fractures underscore their infrequency. These injuries are primarily observed in adolescent males approaching skeletal maturity, often engaged in athletic activities, particularly those involving jumping [1]. This distinct fracture pattern involves the avulsion of the proximal tibial apophysis and, in some instances, extends into the patellar tendon, making it imperative for clinicians to address concomitant injuries when treating these injuries operatively [2].

The existing classification systems, exemplified by the Ogden classification, have historically played a pivotal role in guiding treatment decisions based on lateral radiographs [1]. The Ogden classification system serves as a valuable tool for categorizing tibial tubercle fractures, offering a structured approach to assess the severity of these injuries and guide clinical decision-making [2]. This classification, originally derived from the Watson-Jones system, has undergone modifications to better capture the nuances of tibial tubercle fractures [2]. The Ogden classification comprises five main types, each reflecting distinct patterns of displacement. Type I is a fracture of the secondary ossification center near the insertion of the patellar tendon. Type II fractures propagate proximal between primary and secondary ossification centers. Type III represents a coronal fracture extending posteriorly to cross the primary ossification center. Type IV are fractures through the entire proximal tibial physis and type V injuries are periosteal sleeve avulsion of the extensor mechanism from the secondary ossification center [3]. While the Ogden classification offers a valuable framework for initial assessment based on lateral radiographs, its limitations in fully characterizing the three-dimensional anatomy and potential for underestimating intra-articular involvement underscore the importance of intra-articular visualization during surgical approaches in those injuries that meet indications for operative intervention [4].

For nondisplaced tibial tubercle fractures, treatment typically involves using a brace, cylinder cast, or long leg cast for a period of four to six weeks [5]. In cases of displaced fractures, operative intervention is necessary, typically through screw fixation to the tibial metaphysis [6]. However, if the fragment is substantially comminuted, suture fixation via drill holes or suture anchors may be required. Achieving an anatomic reduction is crucial in cases of displaced fragments, and in instances of greater displacement, there may be a need for repairing the disrupted patellar tendon insertion and extensor retinaculum, with potential additional soft tissue repair [7]. Classically, the portion of the patellar tendon that is avulsed from the tibia distally was either ignored or reattached to the periosteum with suture [8]. In this paper, we describe a method and provide two case examples of open reduction and internal fixation (ORIF) of tibial tubercle fractures with suture anchor repair of the distal patellar tendon avulsion.

## Case presentation

**Figure 1:**
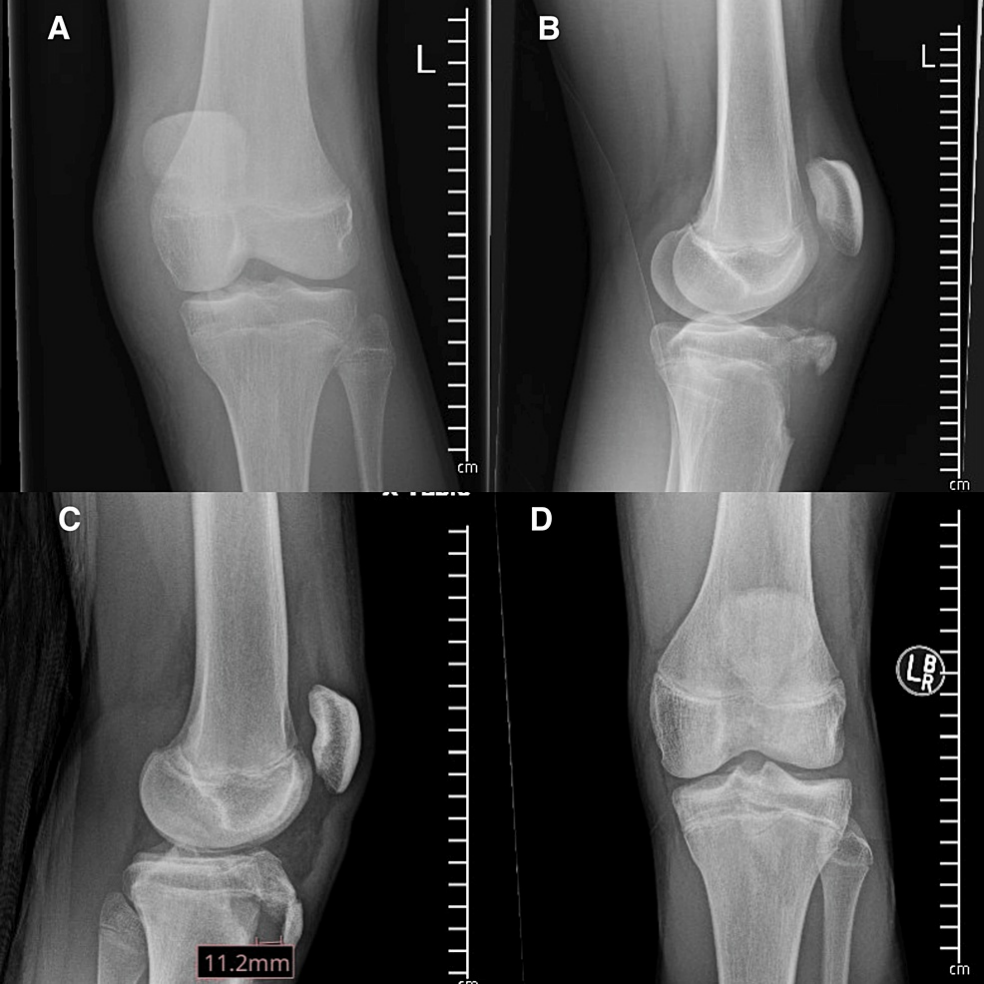
Injury radiographs from case patients: (A) AP radiograph of the knee in patient 1; (B) lateral radiograph of the knee in patient 1; (C) AP radiograph of the knee in patient 2; and (D) lateral radiograph of the knee in patient 2. AP, anteroposterior

**Figure 2:**
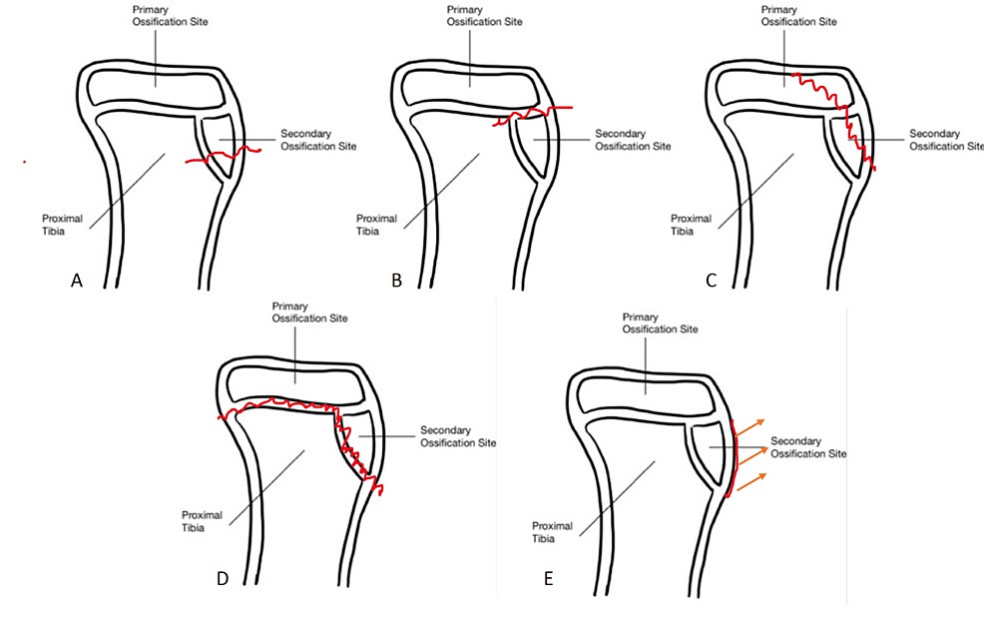
Ogden classification of tibial tubercle fractures. (A)Type I fracture of the secondary ossification center near the insertion of the patellar tendon. (B)Type II fracture propagates proximally between primary and secondary ossification centers. (C) Type III coronal fracture extending posteriorly to cross the primary ossification center. (D) Type IV fracture through the entire proximal tibial physis. (E) Type V periosteal sleeve avulsion of the extensor mechanism from the secondary ossification center. Image credit: Robert Wood.

Both patients were 14-year-old males presenting with the chief complaints of acute knee pain and swelling, accompanied by the inability to bear weight on the injured extremity. The mechanism for both patients was an awkward landing from a layup during a basketball game. Each patient presented to the emergency department, and both were unable to perform a straight leg raise. X-rays were obtained shortly after arrival, and both patients were diagnosed with displaced tibial tubercle fractures (Figure 1). Both fractures were classified as Ogden Type III due to the coronal orientation of the fracture line and the fracture extension through the primary ossification center (Figure 2). After a discussion of both conservative nonoperative management and surgical intervention, both patients elected to move forward with ORIF.

**Figure 3:**
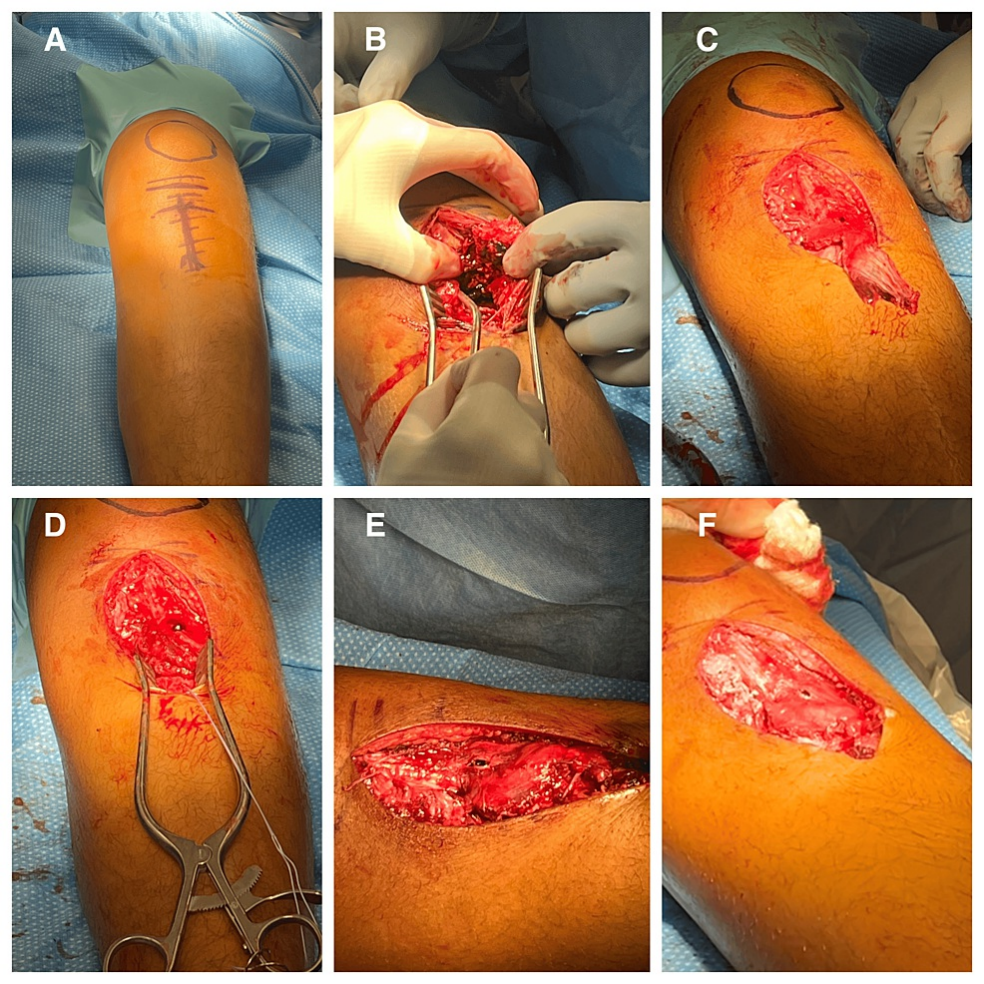
Intraoperative photos demonstrating the described technique. (A) Supine positioning and marking of the planned incision. (B) Open debridement of the fracture site. (C) Debridement of devitalized tissue from the avulsed patellar tendon. (D) Suture anchor placement into the anatomic footprint of the tendon. (E) Placement of Krakow suture and use of tension slide technique. (F) Reduction of the avulsed tendon to the anatomic footprint.

**Figure 4:**
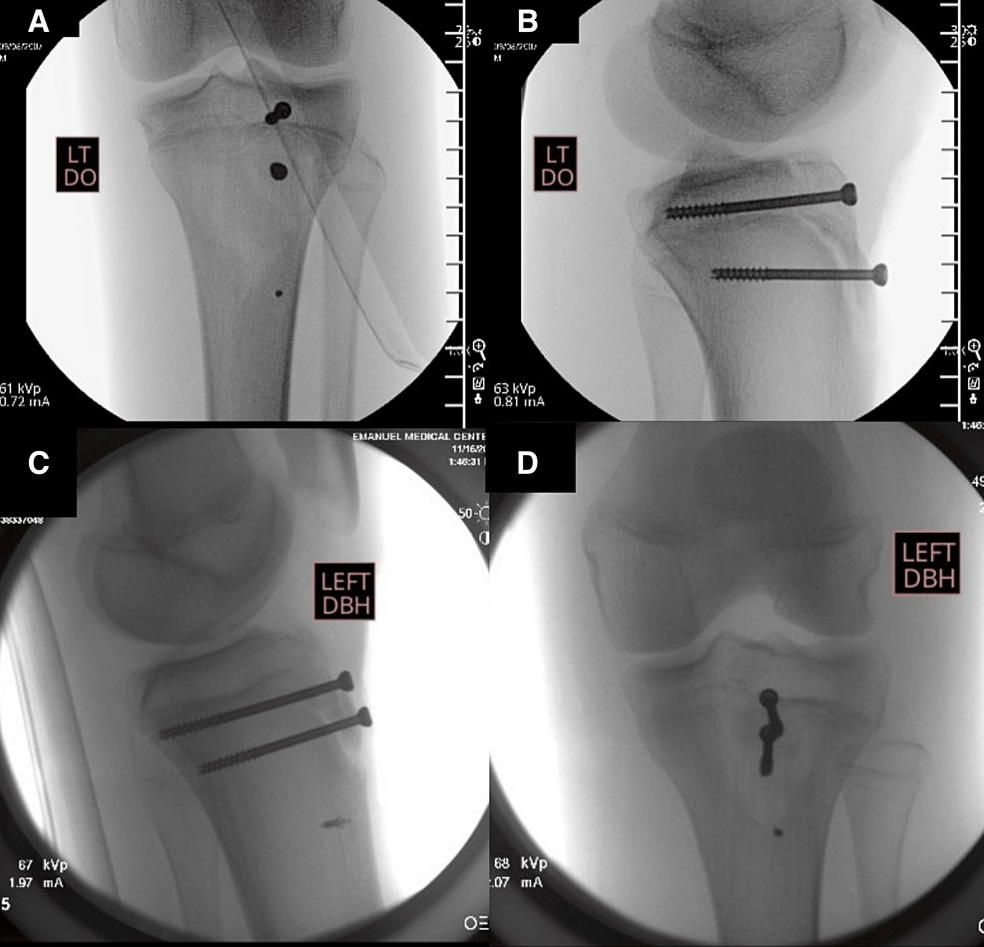
Fluoroscopic intraoperative images from the presented patients demonstrating hardware placement, including percutaneous screws and suture anchor construct. (A) AP of the knee in patient 1; (B) lateral view of the knee in patient 1; (C) lateral view of the knee in patient 2; (D) AP of the knee in patient 2. AP, anteroposterior

Patients were placed supine on a flat-top table with bone foam under the operative leg (Figure 3). A thigh tourniquet was utilized in both cases. A midline incision was made extending from the inferior pole of the patella to just past the tibial tubercle. The paratenon was incised and elevated until the patellar tendon was encountered. The fracture site was then identified, and the fracture hematoma was evacuated. The articular surface and menisci were visualized directly and found to be uninjured in both patients. The fracture site was debrided with curettes and a synovial Ronguer. Manual reduction with a bone tamp was achieved and provisional fixation was maintained with two midline Kirschner wires (K-wires). The K-wires were then over drilled and final fixation was obtained with 4.0 mm cannulated partially threaded lag screws. Attention was then turned to the distal avulsion of the patellar tendon sleeve. Devitalized tissue was debrided off of the distal aspect of the tendon. The anatomic footprint of the avulsed tissue was identified and decorticated to bleeding bone. Then, a 2.4 mm drill was used to drill a pilot hole in the anteromedial tibia in line with the anatomic footprint of the avulsed tendon. Next, a single Mitek G2 Anchor was placed into the pilot hole using a mallet to ensure adequate depth. A locking Krakow stitch was placed through the avulsed tendon tissue and the tendon was pulled back down to its anatomic footprint with a tension slide technique. The retinacular defects were subsequently repaired using 0 Vicryl, and the wound was closed in a standard manner. Fluoroscopic imaging revealed maintained adequate fracture reduction without signs of hardware prominence (Figure 4).

**Figure 5:**
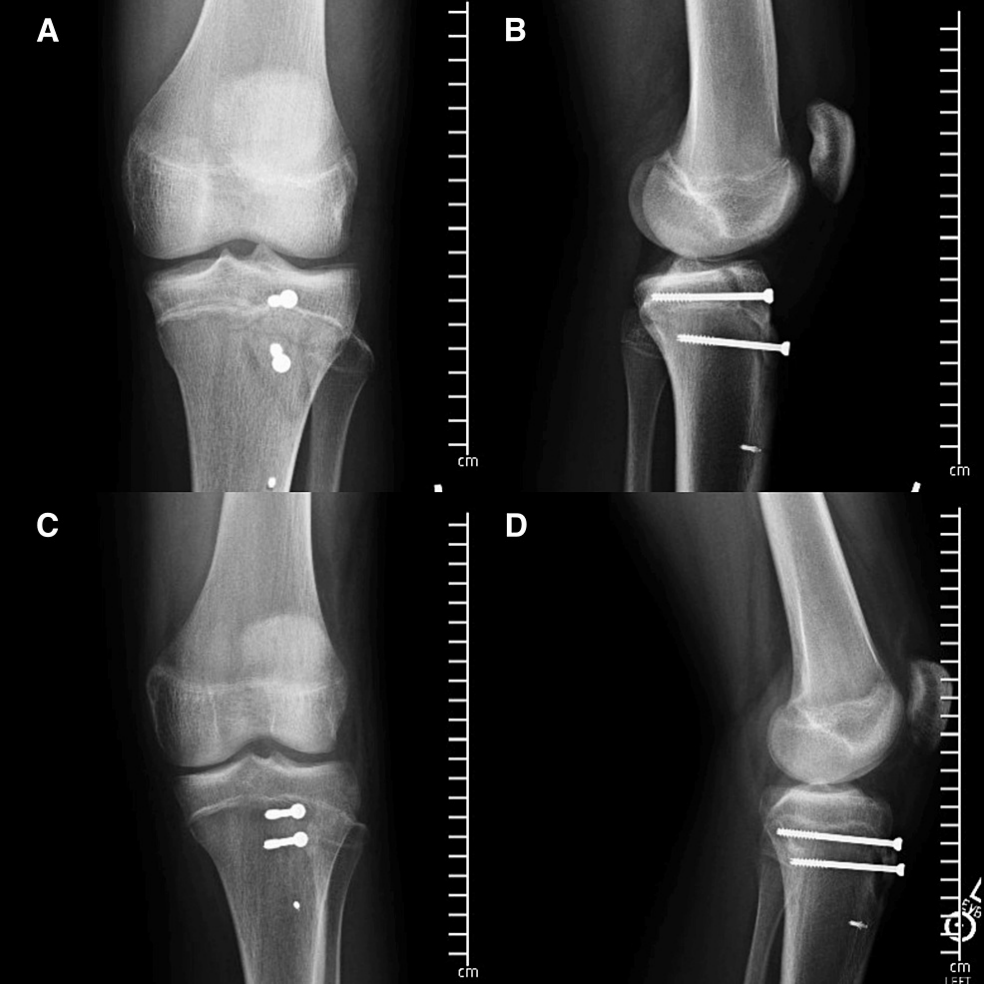
Six-week postoperative images demonstrating a stable appearance of the hardware in the presented patients. (A) AP of the knee in patient 1; (B) lateral view of the knee in patient 1; (C) AP of the knee in patient 2; and (D) lateral view of the knee in patient 2. AP, anteroposterior

Each patient underwent postoperative immobilization with allowance for 0°-30° of knee flexion. Both patients were permitted toe-touch weight-bearing in the immediate postoperative period. Postoperative imaging performed six weeks after surgery revealed maintained fracture reduction without evidence of hardware complication (Figure 5). Immobilization was continued for a total of four weeks with a gradual return to full weight-bearing and range of motion beginning thereafter. At six weeks, patients began formal physical therapy, and both patients were able to return to their previous level of activity and sports participation by four months postoperatively. There were no intraoperative complications, and there was no need for hardware removal at the four-month follow-up mark.

## Discussion

Tibial tubercle fractures are uncommon injuries that occur in the athletic adolescent population [1]. Fractures with significant displacement require surgical intervention and often demonstrate extension of the injury to involve the distal footprint of the patellar tendon [2]. A proposed sequence of this injury in adolescents who sustain tubercle avulsion fractures involves failure of the bony tubercle followed by a rotational moment [9]. This rotation creates tension in the surrounding soft tissues, preventing the translation of the fragment [4]. Subsequent quadriceps contraction further contributes to the process, ultimately resulting in the distal tendon avulsion [9,10]. There is no consensus on the management of this injury, but previous reports have described direct repair to the periosteum with sutures [11]. While this may be suitable, there remains concern regarding the strength of the repair.

In cases of isolated patellar tendon rupture, transosseous tunnels had previously been the standard method utilized for repair regardless of the location of rupture [12]. However, issues with re-rupture rates have led some surgeons to opt for the use of suture anchor constructs. Suture anchor repair has proven to be biomechanically superior to transosseous tunnel fixation in several studies [13]. When compared to the transeosseous tunnel construct, suture anchor repair results in less displacement after cyclic loading, greater construct stiffness, and ultimately a higher load to failure [14]. Our technique employed this biomechanical advantage in repairing the distal avulsed component of the patellar tendon. It provides additional fixation to an unstable injury and represents complete repair of the extensor mechanism that can occur with these injuries. Additionally, both cases remained under the average surgical cost for ORIF of tibial tubercles at our institution and had tourniquet times of less than 90 minutes, which is within the average range of surgical time for this procedure performed at our institution.

## Conclusions

The technique described is a simple and quick method for addressing the distal patellar tendon avulsion component of tibial tubercle fractures. It leverages biomechanical properties to improve upon the traditional method of repair without significantly increasing cost or operative time. The use of a suture anchor may represent a viable method to complete the repair of the extensor mechanism in this injury. Both patients returned to competitive athletics at the high school level at four months postoperatively without issue, and there were no associated hardware complications identified at follow-up.

## References

[1] Frey S, Hosalkar H, Cameron DB, Heath A, David Horn B, Ganley TJ (2008). Tibial tuberosity fractures in adolescents. J Child Orthop.

[2] Reyes CD, Wu W, Pandya NK (2023). Adolescent tibial tubercle fracture: review of outcomes and complications. Curr Rev Musculoskelet Med.

[3] Wiss DA, Schilz JL, Zionts L (1991). Type III fractures of the tibial tubercle in adolescents. J Orthop Trauma.

[4] Pandya NK, Edmonds EW, Roocroft JH, Mubarak SJ (2012). Tibial tubercle fractures: complications, classification, and the need for intra-articular assessment. J Pediatr Orthop.

[5] Checa Betegón P, Arvinius C, Cabadas González MI, Martínez García A, Del Pozo Martín R, Marco Martínez F (2019). Management of pediatric tibial tubercle fractures: is surgical treatment really necessary?. Eur J Orthop Surg Traumatol.

[6] Pretell-Mazzini J, Kelly DM, Sawyer JR, Esteban EM, Spence DD, Warner WC Jr, Beaty JH (2016). Outcomes and complications of tibial tubercle fractures in pediatric patients: a systematic review of the literature. J Pediatr Orthop.

[7] Howarth WR, Gottschalk HP, Hosalkar HS (2011). Tibial tubercle fractures in children with intra-articular involvement: surgical tips for technical ease. J Child Orthop.

[8] Rickert KD, Hedequist D, Bomar JD (2021). Screw fixation of pediatric tibial tubercle fractures. JBJS Essent Surg Tech.

[9] Uppal R, Lyne ED (2007). Tibial tubercle fracture with avulsion of the patellar ligament: a case report. Am J Orthop (Belle Mead NJ).

[10] Pereira AL, Faria ÂRV, Campos TV, Andrade MA, Silva GM (2018). Tibial tubercle fracture associated with distal rupture of the patellar tendon: case report. Rev Bras Ortop.

[11] Wu KC, Ding DC (2013). Tibial tubercle fracture with avulsion of patellar ligament. Formos J Musculoskelet Disord.

[12] Nakashima H, Takahara Y, Uchida Y, Kato H, Itani S, Iwasaki Y (2022). Patellar tendon repair with suture tape augmentation for proximal patellar tendon rupture. Arthrosc Tech.

[13] Bushnell BD, Byram IR, Weinhold PS, Creighton RA (2006). The use of suture anchors in repair of the ruptured patellar tendon: a biomechanical study. Am J Sports Med.

[14] O'Donnell R, Lemme NJ, Marcaccio S, Walsh DF, Shah KN, Owens BD, DeFroda SF (2021). Suture anchor versus transosseous tunnel repair for inferior pole patellar fractures treated with partial patellectomy and tendon advancement: a biomechanical study. Orthop J Sports Med.

